# Applications of Gold Nanoparticles in Nanomedicine: Recent Advances in Vaccines †

**DOI:** 10.3390/molecules22050857

**Published:** 2017-05-22

**Authors:** Sónia Alexandra Correia Carabineiro

**Affiliations:** Laboratório de Catálise e Materiais (LCM), Laboratório Associado LSRE-LCM, Faculdade de Engenharia, Universidade do Porto, 4200-465 Porto, Portugal; scarabin@fe.up.pt; Tel.: +351-22-041-4907

**Keywords:** gold nanoparticles, vaccines, nanomedicine, cancer, AIDS, hepatitis B

## Abstract

Nowadays, gold is used in (nano-)medicine, usually in the form of nanoparticles, due to the solid proofs given of its therapeutic effects on several diseases. Gold also plays an important role in the vaccine field as an adjuvant and a carrier, reducing toxicity, enhancing immunogenic activity, and providing stability in storage. An even brighter golden future is expected for gold applications in this area.

## 1. Introduction

**Figure 1:**
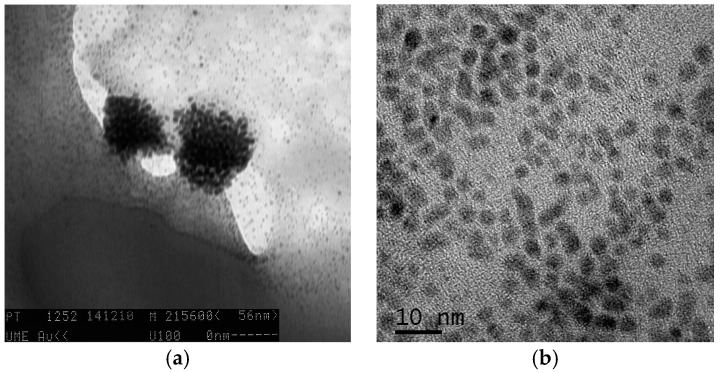
Transmission electron microscopy images (**a**,**b**) of gold nanoparticles in aqueous solution (obtained in a Leo 906 E apparatus at 100 kV).

It is well known that gold is inert in the bulk stable and is only active as a catalyst in the form of nanoparticles [1,2,3,4,5,6,7]. As the name suggests, nanoparticles have small (nano) size and corresponding large surface/volume ratio. Examples are shown in Figure 1. Due to these characteristics, Au nanoparticles can have an important role, not only in catalysis, but also in nanomedicine, suitable for several biological applications. In fact, it has been argued that Au nanoparticles could be used in almost all medical applications, from diagnostics to therapy, prevention, and hygiene [8,9,10,11,12,13,14]. Gold nanoparticles have remarkable properties and can also play an important role in vaccine research for several diseases, as showed in several recent reviews [8,9,15,16,17,18,19,20,21,22,23,24,25,26,27,28,29,30].

**Figure 2:**
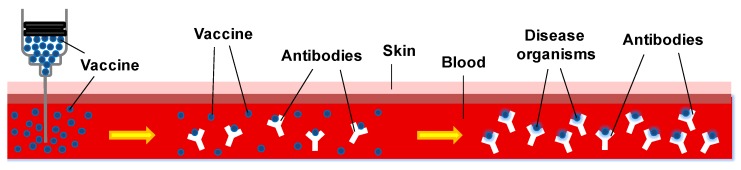
A vaccine in action: the introduction of an antigen of a pathogen in blood stream (**left**) stimulates the production of antibodies against the pathogen (**middle**); so that in case of infection by the actual pathogen (**right**); the immune system is ready to fight the disease (adapted and redrawn from Mayo Clinic Foundation for Medical Education and Research).

Vaccines have been a significant advancement in public health, preventing the death of many millions of people every year [31]. The main purpose of vaccination is to introduce an antigen from a virus or a bacteria that elicit humoral immunity in the body, in order to stimulate the immune system and develop specific immunity (antibodies) against that particular pathogen without causing disease associated with that organism [32]. A general scheme is shown in Figure 2. Most viral vaccines are based on live attenuated or killed viruses (first generation vaccines), while many bacterial vaccines are based on cellular components of micro-organisms, including harmless toxin components (second generation vaccines) [32]. DNA vaccines are third generation vaccines, and are made up of small circular pieces of pathogen DNA (plasmids), genetically engineered to produce specific antigens [33]. Compared to traditional vaccination systems, vaccines made from nanomaterials can have many benefits, including a precise stimulation of the immune response, effective targeting of certain tissue or cells, and biocompatibility [13,16,17,18,19,23,25,26,31,34,35,36,37,38,39,40,41,42,43,44,45].

It is worth noting that intracellular bacteria triggers specific cellular immunity, eliciting CD4 and CD8^+^T cells that recognize processed bacterial antigens. Cellular specific immunity is also elicited by gold nanoparticles vaccines, such as those protecting against listeriosis (see Section 6).

**Scheme 1:**
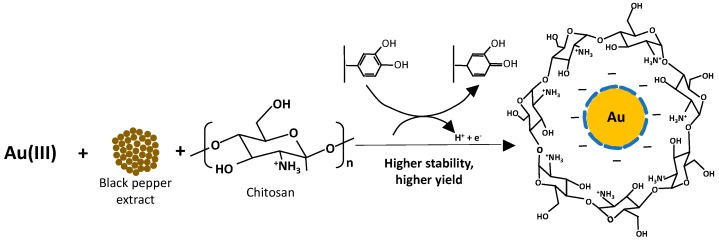
Synthesis of biopolymer-inspired gold nanoparticles reported by Saha et al. (adapted and redrawn from [47]).

Usually nanoparticles are synthesized in colloidal solution by reduction of chloroauric acid (HAuCl_4_) by variations of the so-called Turkevich method [46]. Their characterization is carried out by ultraviolet-visible spectroscopy, their diameters determined by transmission electron microscopy. Then, they can be further functionalized with the desired molecules, according to their specific needs. One example is given in Scheme 1, showing a procedure carried out by Saha et al. [47]. Gold nanoparticles were synthesized in aqueous medium by an ultra-sound assisted green method using black pepper extract and chitosan as reducing agent and a stabilizing agent, respectively. The biopolymer-inspired biogenic method (Scheme 1) was found to be stable and with enhanced bioactivity. The developed nanomaterial boosts the production of reactive oxygen species and misbalances the antioxidant parameters of detoxification enzymes of filarial parasites, such as GST, which besides its immune prophylactic potency, is also a promising candidate vaccine as shown in *S. cervi* and *W. bancrofti* [47].

Gold nanoparticles, in general, have remarkably high surface-to-volume ratio, are biocompatible and inert, and can be easily functionalized with several molecules; thus, they can also play an important role in the vaccine field as adjuvants, reducing toxicity, enhancing immunogenic activity, and providing stability of vaccine in storage, and have great potential as carriers for the development of a great diversity of fully synthetic vaccines [8,9,10,11,12,15,20,21,26,48,49,50,51,52,53,54,55,56,57,58,59,60,61]. Their shape and size can affect immunological responses in vivo and in vitro [51,62,63,64,65]. Moreover, they are able to penetrate blood vessels and tissue barriers and to be targeted to a specific cell by means of specifically functionalized molecules [59]. Moreover, gold nanoparticles can be packaged inside virus-like particles generated by heterologous expression of viral structural genes that are powerful tools in vaccine development [66].

Anionic gold nanoparticles increased the half-life of a green fluorescent protein expressing adenovirus from similar to 48 h to 21 days at 37 °C (from 7 to >30 days at room temperature) [61]. Promising CD8^+^ T cell results were obtained with polyelectrolyte multilayers assembled on gold nanoparticles [67,68]. A positively charged antigen and a negatively charged immuno-adjuvant on gold nanoparticles resulted in a new vaccine platform.

An immunostimulatory nanocomposite (CpG-Au@HBc VLP), designed by self-assembling engineered virus-like particles encapsulating CpG-gold nanoparticle conjugates through electrostatic interactions, showed an increase in CD4^+^ and CD8^+^ T cell numbers, inducing important cellular and humoral immune response [69].

Ultrasmall graphene oxide-supported gold nanoparticles (usGO-Au) were obtained from reduction of chloroauric acid using usGO and then decorated with ovalbumin antigen (OVA) through physical adsorption to obtain usGO-Au@OVA and used immune adjuvants [70]. It was shown that usGO-Au@OVA can efficiently stimulate RAW264.7 cells to secrete tumor necrosis factor (TNF-), a mediator for cellular immune response. usGO-Au@OVA can also promote robust OVA specific antibody response, CD8^+^ T cells proliferation, and secretion of different cytokines.

Fluorescent Au nanoclusters were synthesized using ovalbumin-CpG oligodeoxynucleotides (ODNs) conjugates as templates [71]. The nanoclusters can act as self-vaccines to assist in generation of high immunostimulatory activity.

Gold-based nanovaccines were synthesized using a self-assembling conjugation method [53]. Dendritic cells uptake gold nanovaccines with minimal toxicity and are able to process the vaccine peptides on the particles to stimulate cytotoxic T lymphocytes (CTLs). These high-peptide density Au nanovaccines can stimulate CTLs better than free peptides and have great potential as carriers for various vaccine types [53].

Hydrophilic gold nanodots were used to control lipopolysaccharide assembly to ease the formation of stable endotoxin nanovesicles, which are stable precursors of cubosomes and hexosomes with specific immunological effects that might be useful in vaccine development [72].

Needle-free vaccine delivery systems efficiently transport powdered or particulate DNA and protein vaccines into the epidermal tissue [73,74,75,76]. It can be used to directly transfect antigen presenting cells by formulating DNA or protein vaccines onto 1–3 µm gold particles (particle-mediated immunization). A solid-in-oil dispersion of gold nanorods can also enhance transdermal protein delivery and skin vaccination [77]. Plasmid DNA (pDNA)-coated gold nanoparticles were successfully delivered into ex vivo murine and porcine skin at low inlet pressures using parallel arrays of microchannels [78]. It was shown that full-length pDNA was preserved after each particle preparation and jetting procedures.

Below, several examples are given on the role of gold nanoparticles in the attempts of producing vaccines for several diseases.

## 2. Cancer Vaccines

Cancer is one of the main causes of death worldwide, that can affect people at all ages, even small children and foetuses, the risk usually increasing with age [32,79]. The present treatments consist of surgery, chemotherapy, or radiotherapy, which can have adverse side effects, due to a lack of specificity for tumors [80]. An ideal treatment should aim only at the target tumor cells and have limited detrimental effects on normal cells [32,79].

It was first postulated by Coley in 1928 that the immune system was able to recognize and set a response against tumors [81]. Later, it was shown that immunization of mice with mutated tumor cells could induce a protective anti-tumor immune response against non-immunogenic tumor [32]. This led to cancer immunotherapy research and to the development of cancer vaccines capable of generating an active tumor-specific immune response.

Cancer vaccines have high specificity for tumor cells and long-lasting immunological memory that may safeguard against recurrences, and can be used to either prevent (prophylactic) or treat established cancer (therapeutic) [32,79]. Therapeutic cancer vaccines (also called active immunotherapy) work to enable the immune system of a patient to eradicate cancer cells [82].

Identification of tumor-associated antigens (TAAs) and tumor-specific antigens (TSAs) has led to increased efforts to develop vaccination strategies. Vaccines may be composed of whole cells or cell extracts, genetically modified tumor cells, dendritic cells (DCs) loaded with TAAs, immunization with soluble proteins or synthetic peptides, recombinant viruses or bacteria encoding tumor-associated antigens, and plasmid DNA-encoding TSAs or TAAs in conjunction with appropriate immunomodulators [32,79]. All of these vaccination approaches aim to induce specific immunological responses and are localized into TAAs, destroying only the tumor cells and leaving the majority of other cells of the body undamaged.

**Figure 3:**
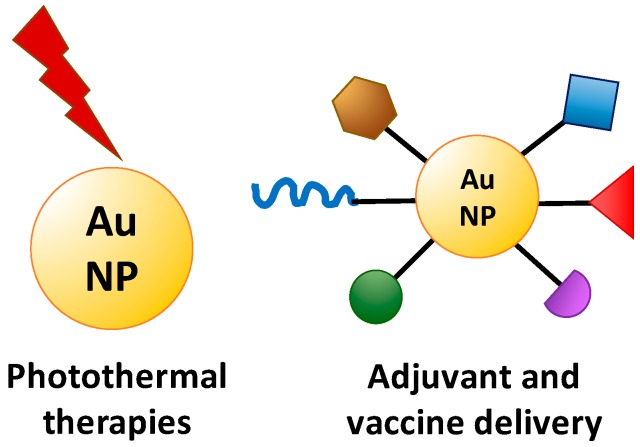
Applications of gold nanoparticles in cancer immunotherapy (adapted and redrawn from [87]).

An efficient delivery to cells is needed and gold nanoparticles have proved to be excellent carriers [83,84,85,86,87,88,89]. Gold nanoparticles enabled efficient antigen delivery to dendritic cells and then activated the cells to facilitate cross-presentation, inducing antigen-specific cytotoxic T-lymphocyte responses for effective cancer therapy [88]. Moreover, they show several advantages over other nanoparticulate-based carriers, such as the ease with which they control their size for different applications, the variety of antigens and adjuvants that can be easily linked to and displayed on their surface, the fact that they can be detected using non-invasive imaging techniques, providing clinicians with information on where or whether the vaccines have been delivered, which helps to predict or evaluate therapeutic efficacy, and the biocompatibility and non-toxicity of gold [86,87,90]. Au nanoparticle immunotherapies are well suited for synergistic combination therapy with existing cancer therapies such as photothermal ablation [59,87]. All these features suggest that gold nanoparticle-based antigen delivery systems may be a useful vaccine technology that is able to prevent and/or treat cancer. The several functionalities of gold nanoparticles make them promising vehicles for immune therapies, especially for combinatorial treatment approaches that target multiple immune pathways (Figure 3).

A polymerizable version of the Tn-antigen glycan was synthesized and the polymers were conjugated to gold nanoparticles, producing nanoscale glycoconjugates [91]. These nanomaterials generated a strong and long-lasting production of antibodies, selective to the Tn-antigen glycan and cross-reactive toward mucin proteins displaying Tn, and thus represent a simple approach to the synthesis of anticancer vaccines.

Gold nanoparticles obtained by a modified Turkevich method were functionalized with mucin-1 (MUC-1) glycoprotein using Clealand’s reagent [82]. (MUC-1 is involved in fundamental biological processes that can be found over-expressed and with a clearly changed glycan pattern on epithelial tumor cells, and thus is a promising target structure in the search for effective carbohydrate-based cancer vaccines and immunotherapeutics [58]). The obtained Au-MUC-1 nano-construction has been shown to be an important macrophage activator, leading to the release of cytokines such as TNF-alpha, IL-6, IL-10, and IL-12 on peritoneal macrophages isolated from mice [82].

Other authors also reported on the synthesis of MUC1-glycopeptide antigens and their coupling to gold nanoparticles of different sizes and developed a new dot-blot immunoassay to test the potential antigen-antibody binding [58]. A glycopeptide sequence derived from MUC-1 glycoprotein and the T-cell epitope P30 sequence were immobilized gold nanoparticles attached to polyethylene glycol chains [92]. After immunization, mice showed significant MHC-II mediated immune responses, and their antisera were able to recognize human MCF-7 breast cancer cells. Gold nanoparticles designed this way have the potential to be used in the development of anticancer vaccines.

Gold nanoparticles proved to be efficient in facilitating the delivery of the ovalbumin (OVA) peptide antigen and the CpG adjuvant and enhance their therapeutic effect in a B16-OVA tumor model. Gold nanoparticle-mediated OVA peptide delivery can have important therapeutic benefits without the need of an adjuvant, showing that gold nanoparticles are effective peptide vaccine carriers, having the potential to allow the use of lower and safer adjuvant doses during vaccination [93].

## 3. HIV Vaccines

**Figure 4:**
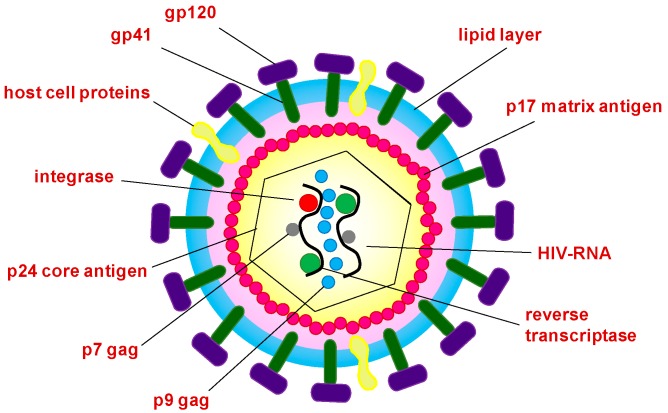
HIV virus structure anatomy (from Health Medicine, adapted and redrawn from http://www.clipartkid.com/hiv-cliparts/).

The use of gold nanoparticles in HIV vaccine development has been recently reviewed by several authors [94]. After more than 30 years since the discovery of HIV in 1983, no effective vaccine is yet available [24,95,96,97]. Some histocompatibility complex molecules expressed on the surface of HIV are potential targets for neutralizing antibodies [98], as shown in Figure 4. Among the few broadly neutralizing HIV monoclonal antibodies, 2G12 is the only carbohydrate-directed that is able to recognize a cluster of high-mannose glycans on the viral envelope glycoprotein gp120 [95]. This type of glycan is thus envisaged as a target to develop an HIV vaccine capable of eliciting 2G12-like antibodies. Gold nanoparticles coated with self-assembled monolayers of synthetic oligomannosides, which are present in gp120, are able to bind 2G12 with high affinity and to interfere with 2G12/gp120 binding [95]. Thiol-terminated oligosaccharides have been attached on gold nanoparticles and have been used in attempts to develop an HIV vaccine [99].

Two nanometer gold glyconanoparticles were coated with synthetic partial structures of several mannosides [100]. The assembly of the antennas of the gp120 high-mannose type glycan on gold glyconanoparticles provided superior binding to the anti-HIV antibody 2G12, which could help in the design of a carbohydrate-based vaccine against HIV.

It was shown that conjugation to negatively charged gold glyconanoparticles could stabilize either the alpha-helix or beta-strand conformation of the third variable region (V3 peptide) of the HIV-1 gp120 [101]. The peptide on the nanoparticles shows more stability toward peptidase degradation compared to the free peptide. V3 beta-glyconanoparticles produce antibodies in rabbits that recognize a recombinant gp120, and the serum showed consistent neutralizing activity. These results potentially allow for the design of new fully synthetic HIV vaccine candidates [101].

Gold nanorods modified with poly(diallydimethylammonium chloride) or polyethyleneimine can significantly promote cellular and humoral immunity, as well as T-cells proliferation, through activating antigen-presenting cells, when compared to naked HIV envelope plasmid DNA treatment in vivo [102]. This makes gold nanorods promising DNA vaccine adjuvants for HIV treatment.

## 4. Encephalitis Vaccines

The use of gold nanoparticles in designing vaccines against tick-borne encephalitis (TBE) was proposed for the first time by Demenev et al. [103]. The vaccine was prepared by conjugating colloid gold with a soluble TBE antigen. In animals immunized with the experimental vaccine, the protection coefficient and mean survival time were, respectively, 1.3–1.5 times and 10–30% higher than in mice immunized with a commercial vaccine. Moreover, the mean survival time was 1.2–1.7 times longer in animals injected with antibodies from mice immunized with the experimental vaccine, compared with the commercial one.

In a more recent study, colloidal gold particles were used as carriers of protein antigen of the capsid of the TBE virus in the antiviral vaccine [104]. After two inoculations, the mice in that study were found to resist challenge with 100,000 times the 50% lethal dose (LD50) of JEV (Beijing-1 strain), even when immunized with a relatively small dose of 0.5 mug of plasmid DNA.

Japanese B encephalitis vaccine is an important vaccine to prevent this serious mosquito-borne disease caused by the virus [105,106]. There are some semiquantitative methods to determine this vaccine, such as plaque forming unit and the animal testing, but they have low sensitivity, are time-consuming, and have a high cost. A label-free amperometric immunosensor for fast and sensitive assay of Japanese B encephalitis vaccine was reported with a specific response in the range 1.1 × 10^−8^ to 1.9 × 10^−6^ lg·pfu/mL (pfu/mL is plaque forming unit and lg is common logarithm) with a detection limit of 6 × 10^−9^ lg·pfu/mL [105]. An immunosensor based on the immobilization of antiserum of Japanese B encephalitis on a gold electrode modified by [nano-Au/Co(bipyridine)_3_^3+^]_2_/nano-Au/l-cysteine has also been reported [106]. It exhibited fast potentiometric high sensitivity and long-term stability. These works are promising test methods for biological products.

## 5. Hepatitis Vaccines

**Figure 5:**
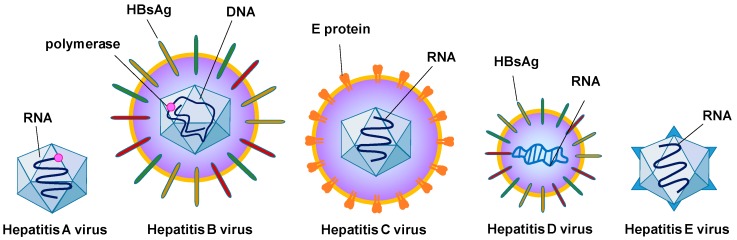
Comparison of viruses from the different types of hepatitis (adapted and redrawn from http://www.123rf.com/profile_alila).

Hepatitis is inflammation of the liver that may originate a yellow color in the skin and eyes and abdominal pain, among other symptoms that can be caused by viruses or from the abuse of alcohol and certain medications. There are several types of viral hepatitis: types A, B, C, D, and E. Hepatitis A and E are often spread by contaminated food and water, while hepatitis B is mainly sexually transmitted but may pass from mother to child during pregnancy. Types B and C are usually spread through infected blood. Hepatitis D can only infect people already infected with hepatitis B. Figure 5 shows a comparison between the viruses.

Concerning hepatitis B vaccine research, HBsAg-DNA can be introduced into the host by intramuscular or intradermal route using a needle with syringe. An alternative is using gene-gun, Biolistic^®^, PowderJectTM, Accell^®^, or particle-mediated DNA delivery, when DNA-coated microscopic gold particles are transported using a needle-free device directly into the epidermic cells [75]. Micron-size gold particles were used as particulate adjuvants and coinjected intradermally with plasmid DNA encoding the HBsAg into mice [107,108]. The presence of gold particles accelerated the antibody response significantly, increased the percentage of responding animals, and shortened the time taken to reach maximal antibody titers by two weeks. These immunizations were effective in protecting mice against tumor challenge with cancer cells, expressing HBsAg as a surrogate cancer antigen. Good results were also obtained with minipigs [109].

Electrically activated plasmonic Au nanoparticles can drive vibrational and dipole-like oscillations that are able to disrupt nearby cell membranes, allowing enhanced cell poration and facilitating the uptake of a model hepatitis C virus DNA vaccine was recently reported [110]. Immunized mice showed up to 100-fold higher gene expression compared to control treatments (without nanoparticles) and exhibited significantly increased levels of both antibody and cellular immune responses.

A new method of preparation of a vaccine for hepatitis E (HEVA) using in situ easy growth of gold clusters in the vaccine was recently reported [111]. The prepared HEVA/Au enhances its immune responses in vivo and reduces the potential toxicity of HEVA. The fluorescence of gold clusters enables the HEVA to be traceable, which may open a way to track the dynamic behavior of vaccines and further help to optimize an individual therapeutic regimen for immunotherapy [111].

## 6. Other Vaccines

Gold has been used in several other vaccines. Some examples are reported below.

Gold nanoparticles were conjugated to a synthetic peptide of the VP1 capsid protein of the foot-and-mouth disease virus, which causes an acute, highly contagious infection of domestic and wild animals, transmittable to humans, for which the existing vaccines are not much effective [112]. The resulting conjugate (with or without the use of complete Freund’s adjuvant) was compared to a commercial vaccine and to the native peptide, in immunization of guinea pigs. The titer and sensitivity of the antibodies were maximal for the combination comprising the nanoparticle-peptide conjugate and complete Freund’s adjuvant. Gold nanoparticles were evaluated as vaccine carriers for enhancing the antibody response against a resembling foot-and-mouth disease virus peptide [62]. Particles with 8–17 nm in diameter stimulated the highest antibody levels and accumulated at the highest numbers in the spleen of mice.

Recently, the potential of chitosan functionalized gold nanoparticles (CsAuNPs) for the transmucosal delivery of tetanus toxoid vaccine was shown [63,113,114]. Excellent stability was found for the formulation at recommended storage conditions. CsAuNPs were also used as a carrier for the tetanus toxoid (TT) antigen along with the immunostimulant *Quillaja saponaria* extract (QS) [115]. The synthesized CsAuNPs were spherical, around 40 nm in size and conferred protection to TT against gastric hydrolysis. TT-QS-CsAuNPs induced up to 28-fold immune responses compared to TT and TT-QS controls, after oral administration in mice. The delivery of TT and QS with functionalized CsAuNPs might be a good approach for oral vaccine delivery [115].

Gold glyconanoparticles proved to be good carriers for a synthetic *Streptococcus pneumoniae* type 14 conjugate vaccine [50]. A gold nanorod construct that displayed the major protective antigen of the respiratory syncytial virus (which causes pneumonia and wheezing in children and the elderly), the fusion protein (F) was reported, which can be a candidate vaccine preparation by the covalent attachment of viral protein using a layer-by-layer approach [116].

**Figure 6:**
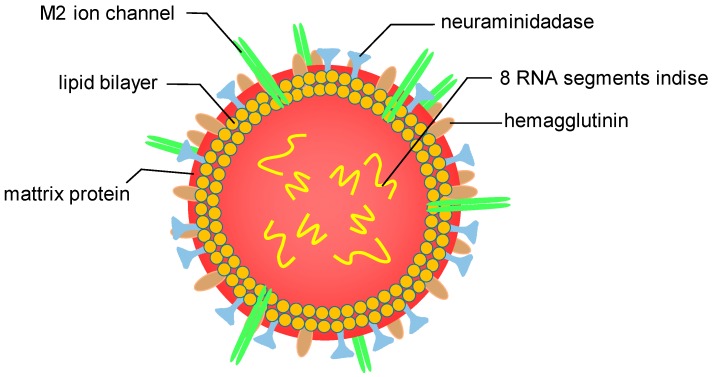
Anatomy of the flu virus (adapted and redrawn from http://www.euroclinix.net).

Gold nanoparticles of 12 nm were conjugated to the matrix 2 protein (M2e) of influenza A virus M2e through thiol–gold interactions [117]. This virus (Figure 6) is often responsible for seasonal epidemics and is also a high risk for pandemics [118]. Vaccination of mice with M2e-Au conjugates induced M2e-specific IgG serum antibodies, which significantly improved by addition of CpG as adjuvant, as mice immunized that way were fully protected against lethal PR8 [117]. The same authors also showed that the inclusion of excess soluble M2e antigen along with M2e immobilized on Au nanoparticles is vital for inducing high levels of antibody response, and in providing complete protection [118].

A recent study showed that animals immunized with a transmissible gastroenteritis virus, conjugated with colloidal gold nanoparticles, produced antibodies with a higher titer than those produced in response to the native virus [119].

A gold nanoparticle antigen delivery approach was used together with a novel polysaccharide nanoparticulate adjuvant, and an effective T-cell vaccine was developed providing protection in animal models of listeriosis (a fatal infection for fetuses and newborns that causes meningitis and cutaneous lesions) [120]. This antigen delivery approach against listeriosis, using gold nanoparticles and bacterial peptides, elicits protective cellular immunity, independent of the adjuvant used, either as a carbohydrate such as Advax [121] or a TLR-4 agonist such as DIO-1 [120]. Gold glyconanoparticles loaded with listeriolysin peptide 91-99 (LLO91-99) or glyceraldehyde-3-phosphate dehydrogenase 1-22 peptide (GAPDH(1-22)) were successfully administrated to pregnant female mice as a vaccination for this disease [122]. Neonates from vaccinated mothers were free of bacteria and healthy, while non-vaccinated mice showed clear brain affections and cutaneous diminishment of melanocytes.

Amphiphilic surface ligand-coated Au nanoparticles able to target myeloid dendritic cells in lymph nodes were used as a peptide antigen carrier, increasing the efficacy of a model vaccine in B16-OVA melanoma mouse models [123].

Gold nanoparticles were coated with the F1-antigen of *Yersinia pestis* (bacterium responsible for the plague), using *N*-hydroxysuccinimide and *N*-(3-dimethylaminopropyl)-*N*′-ethylcarbodiimide hydrochloride coupling chemistry, and administrated to mice, triggering an IgG antibody response to the F1-antigen [124]. The sera raised against F1-antigen coupled to Au nanoparticles were able to competitively bind to recombinant F1-antigen, displacing protective macaque sera.

Target antigens identified in *Plasmodium falciparum*, the parasite responsible for malaria, include surface proteins Pfs230 and Pfs48/45 in male and female gametocytes and Pfs25 expressed in zygotes and ookinetes. The codon-harmonized recombinant Pfs25 in *Escherichia coil* (CHrPfs25) is able to produce malaria transmission-blocking antibodies in mice. CHrPfs25 was also investigated, with gold nanoparticles of different shapes, size and physicochemical properties as adjuvants for induction of transmission blocking immunity, causing transmission blocking antibodies [125]. These results show that gold nanoparticle-based formulations can be developed as nanovaccines to enhance the immunogenicity of vaccine antigens.

Gold nanoparticles were conjugated to N-terminal domains of *Pseudomonas aeruginosa* flagellin recombinant protein through direct interaction of thiol molecules of the cysteines with gold and formation of Au–S bond [126]. Mice that received AuNP-flagellin((1-161)) with Freund’s adjuvant produced high titers of anti-flagellin((1-161)) antibodies that effectively recognized the native flagellin on the bacteria.

Synthetic virus-like particles (sVLPs) were prepared by incubating 100 nm gold nanoparticles in a solution containing an avian *coronavirus* spike protein [127]. After removing the free proteins, antigen-laden particles were recovered and showed morphological semblance to natural viral particles. Vaccination with the sVLPs showed enhanced lymphatic antigen delivery, stronger antibody titers, increased splenic T-cell response, and reduced infection-associated symptoms, and provided superior antiviral protection when compared to a commercial whole inactivated virus vaccine [127].

*Burkholderia mallei* are bacteria responsible for the glanders disease, which is recently classified as a Tier 1 agent, since they can be weaponized for aerosol release, cause high mortality rates, and show multi-drug resistance, and for which there is so far no vaccine available [128]. Gold nanoparticles were covalently coupled with one of three different protein carriers (TetHc, Hcp1, and FliC) followed by conjugation to lipopolysaccharide (LPS) generated significantly higher antibody titers compared with LPS alone. In another study, a gold nanoparticle glycoconjugate composed of *Burkholderia thailandensis* LPS conjugated to FliC was evaluated for the first time as a candidate vaccine for glanders on rhesus macaques (*Macaca mulatta*) [57].

It has recently been shown that the *Pichia pastoris* yeast expression system was adequate for the production of recombinant-truncated proteins, and their apparent bioactivity suggests that tORF25, tORF25C, and tORF25D are potential candidate vaccines against *Cyprinid herpes virus 2* infection. Found in farmed gibel carp *Carassius gibelio*, it is an infectious disease that recently emerged in China that has been troubling the aquaculture industry [129].

Colloidal gold-labelled immunoglobulin was used to confirm Rv1268c protein localization on *Mycobacterium tuberculosis* [130]. These results, along with those obtained for other proteins, might lead to the discovery of select peptides that could have the potential to be included in a subunit-based vaccine for tuberculosis, an infectious disease that remains lethal around the world.

Biocompatible gold nanoparticles were complexed with anti-dengue virus small interfering RNAs (siRNA), and were able to enhance the siRNA delivery and stability, becoming a novel strategy against a virus that causes flu-like symptoms, haemorrhagic fever, and death [131].

*Escherichia coli* as a model pathogen for the design of an antibacterial vaccine [132]. The bacterial outer membrane vesicles were collected and successfully coated onto gold nanoparticles with a 30 nm diameter. The resulting bacterial membrane-coated gold nanoparticles, when injected subcutaneously, induced rapid activation and maturation of dendritic cells in the lymph nodes of the vaccinated mice, generated durable antibody responses, and induced a high production of interferon gamma and interleukin-17, but not interleukin-4, indicating its capability of generating strong Th1- and Th17-biased cell responses against the used bacteria.

A monoclonal antibody raised against human cytomegalovirus (CMV, a herpes virus) surface glycoprotein (gB) was chemically conjugated with gold nanoparticles [133]. The gB-gold nanoparticles blocked viral replication, virus-induced cytopathogenic effects, and virus spread in cell culture without inducing cytotoxicity, and cells treated with them gained resistance to CMV infection.

## 7. Future Prospects

As discussed above, research on gold has indicated its potential for several applications in nanomedicine and its (bio)medical applications, including vaccines, and, as shown in this review, has been increasing. The overall conclusion is that the potential of gold for stimulating research in vaccines is considerable. The results will certainly lead to more practical and commercial applications, the full extent of which has yet to be envisaged. Certainly, the use of gold carries added costs. As newer and more expensive vaccines are introduced and attempted to reach people of different ages and in new settings, the logistics systems must be strengthened and optimized, as recently highlighted and reviewed by Zaffran et al. [134].

## References

[1] Grisel R., Weststrate K.J., Gluhoi A., Nieuwenhuys B.E. (2002). Catalysis by gold nanoparticles. Gold Bull..

[2] Haruta A. (2003). When gold is not noble: Catalysis by nanoparticles. Chem. Rec..

[3] Hashmi A.S.K., Hutchings G.J. (2006). Gold catalysis. Angew. Chem. Int. Ed..

[4] Bond G.C., Louis C., Thompson D.T. (2006). Catalysis by Gold.

[5] Carabineiro S.A.C., Thompson D.T., Heiz E.U., Landman U. (2007). Catalytic Applications for Gold Nanotechnology. Nanocatalysis.

[6] Carabineiro S.A.C., Thompson D.T., Corti C., Holliday R. (2010). Gold Catalysis. Gold: Science and Applications.

[7] Priecel P., Salami H.A., Padilla R.H., Zhong Z.Y., Lopez-Sanchez J.A. (2016). Anisotropic gold nanoparticles: Preparation and applications in catalysis. Chin. J. Catal..

[8] Dykman L.A., Khlebtsov N.G. (2011). Gold Nanoparticles in Biology and Medicine: Recent Advances and Prospects. Acta Nat..

[9] Dykman L., Khlebtsov N. (2012). Gold nanoparticles in biomedical applications: Recent advances and perspectives. Chem. Soc. Rev..

[10] Shah M., Badwaik V.D., Dakshinamurthy R. (2014). Biological Applications of Gold Nanoparticles. J. Nanosci. Nanotechnol..

[11] Maughan C.N., Preston S.G., Williams G.R. (2015). Particulate inorganic adjuvants: Recent developments and future outlook. J. Pharm. Pharmacol..

[12] Versiani A.F., Andrade L.M., Martins E.M.N., Scalzo S., Geraldo J.M., Chaves C.R., Ferreira D.C., Ladeira M., Guatimosim S., Ladeira L.O. (2016). Gold nanoparticles and their applications in biomedicine. Future Virol..

[13] Robles-Garcia M.A., Rodriguez-Felix F., Marquez-Rios E., Aguilar J.A., Barrera-Rodriguez A., Aguilar J., Ruiz-Cruz S., Del-Toro-Sanchez C.L. (2016). Applications of Nanotechnology in the Agriculture, Food, and Pharmaceuticals. J. Nanosci. Nanotechnol..

[14] Klinman D.M., Sato T., Shimosato T. (2016). Use of nanoparticles to deliver immunomodulatory oligonucleotides. Wiley Interdiscip. Rev. Nanomed. Nanobiotechnol..

[15] Fujita Y., Taguchi H. (2011). Current status of multiple antigen-presenting peptide vaccine systems: Application of organic and inorganic nanoparticles. Chem. Cent. J..

[16] Gregory A.E., Titball R., Williamson D. (2013). Vaccine delivery using nanoparticles. Front. Cell. Infect. Microbiol..

[17] Zhu M.T., Wang R.F., Nie G.J. (2014). Applications of nanomaterials as vaccine adjuvants. Hum. Vaccines Immunother..

[18] Marasini N., Skwarczynski M., Toth I. (2014). Oral delivery of nanoparticle-based vaccines. Expert Rev. Vaccines.

[19] Irvine D.J., Hanson M.C., Rakhra K., Tokatlian T. (2015). Synthetic Nanoparticles for Vaccines and Immunotherapy. Chem. Rev..

[20] Alberto J., Salazar G., Gonzalez-Ortega O., Rosales-Mendoza S. (2015). Gold nanoparticles and vaccine development. Expert Rev. Vaccines.

[21] Tao Y., Zhang Y., Ju E.G., Ren H., Ren J.S. (2015). Gold nanocluster-based vaccines for dual-delivery of antigens and immunostimulatory oligonucleotides. Nanoscale.

[22] Comber J.D., Bamezai A. (2015). Gold nanoparticles (AuNPs): A new frontier in vaccine delivery. J. Nanomed. Biotherap. Discov..

[23] Yang L., Li W., Kirberger M., Liao W.Z., Ren J.Y. (2016). Design of nanomaterial based systems for novel vaccine development. Biomat. Sci..

[24] Wang Z.Y., Qin C.J., Hu J., Guo X.Q., Yin J. (2016). Recent advances in synthetic carbohydrate-based human immunodeficiency virus vaccines. Virol. Sin..

[25] Torres-Sangiao E., Holban A.M., Gestal M.C. (2016). Advanced Nanobiomaterials: Vaccines, Diagnosis and Treatment of Infectious Diseases. Molecules.

[26] Vartak A., Sucheck S.J. (2016). Recent Advances in Subunit Vaccine Carriers. Vaccines.

[27] Sun B., Xia T. (2016). Nanomaterial-based vaccine adjuvants. J. Mater. Chem. B.

[28] Ilinskaya A.N., Dobrovolskaia M.A. (2016). Understanding the immunogenicity and antigenicity of nanomaterials: Past, present and future. Toxicol. Appl. Pharmacol..

[29] Dykman L.A., Khlebtsov N.G. (2017). Immunological properties of gold nanoparticles. Chem. Sci..

[30] Marques Neto L.M., Kipnis A., Junqueira-Kipnis A.P. (2017). Role of metallic nanoparticles in vaccinology: Implications for infectious disease vaccine development. Front. Immunol..

[31] Karch C.P., Burkhard P. (2016). Vaccine technologies: From whole organisms to rationally designed protein assemblies. Biochem. Pharmacol..

[32] Aly H.A.A. (2012). Review: Cancer therapy and vaccination. J. Immunol. Methods.

[33] Robinson H.L. (2000). DNA vaccines. Clin. Microbiol. Newsl..

[34] Lico C., Schoubben A., Baschieri S., Blasi P., Santi L. (2013). Nanoparticles in Biomedicine: New Insights from Plant Viruses. Curr. Med. Chem..

[35] Zaman M., Good M.F., Toth I. (2013). Nanovaccines and their mode of action. Methods.

[36] Roy R., Shiao T.C., Rittenhouse-Olson K. (2013). Glycodendrimers: versatile tools for nanotechnology. Braz. J. Pharm. Sci..

[37] Liu Y., Xu Y.Y., Tian Y., Chen C.Y., Wang C., Jiang X.Y. (2014). Functional Nanomaterials Can Optimize the Efficacy of Vaccines. Small.

[38] Zhu X., Radovic-Moreno A.F., Wu J., Langer R., Shi J.J. (2014). Nanomedicine in the management of microbial infection—Overview and perspectives. Nano Today.

[39] Paliwal R., Babu R.J., Palakurthi S. (2014). Nanomedicine Scale-up Technologies: Feasibilities and Challenges. AAPS PharmSciTech.

[40] Prashant C.K., Kumar M., Dinda A.K. (2014). Nanoparticle Based Tailoring of Adjuvant Function: The Role in Vaccine Development. J. Biomed. Nanotechnol..

[41] Sokolova V., Westendorf A.M., Buer J., Uberla K., Epple M. (2015). The potential of nanoparticles for the immunization against viral infections. J. Mat. Chem. B.

[42] Hartwell B.L., Antunez L., Sullivan B.P., Thati S., Sestak J.O., Berkland C. (2015). Multivalent Nanomaterials: Learning from Vaccines and Progressing to Antigen-Specific Immunotherapies. J. Pharm. Sci..

[43] Adak A.K., Li B.Y., Lin C.C. (2015). Advances in multifunctional glycosylated nanomaterials: Preparation and applications in glycoscience. Carbohydr. Res..

[44] Fang R.N.H., Zhang L.F., Prausnitz J.M. (2016). Nanoparticle-Based Modulation of the Immune System. Annual Review of Chemical and Biomolecular Engineering.

[45] Cunha-Matos C.A., Millington O.R., Wark A.W., Zagnoni M. (2016). Real-time assessment of nanoparticle-mediated antigen delivery and cell response. Lab Chip.

[46] Turkevich J., Stevenson P.C., Hillier J. (1951). A study of the nucleation and growth processes in the synthesis of colloidal gold. Discuss. Faraday Soc..

[47] Saha S.K., Roy P., Mondal M.K., Roy D., Gayen P., Chowdhury P., Babu S.P.S. (2017). Development of chitosan based gold nanomaterial as an efficient antifilarial agent: A mechanistic approach. Carbohydr. Polym..

[48] Dykman L.A., Bogatyrev V.A., Staroverov S.A., Semenov S.V. (2003). Gold Colloidal Solution is Used as an Adjuvant, Provides Reducing Toxicity of Vaccines, to Enhance Their Immunogenic Activity and to Provide Stability of Vaccine in Storage.

[49] Yum J.S., Ahn B.C., Moon H.M. (2007). New Adjuvant Composition Comprising Colloidal Gold, Used for Therapeutic Vaccine or for Inducing Immune Response.

[50] Safari D., Marradi M., Chiodo F., Dekker H.A.T., Shan Y.L., Adamo R., Oscarson S., Rijkers G.T., Lahmann M., Kamerling J.P. (2012). Gold nanoparticles as carriers for a synthetic Streptococcus pneumoniae type 14 conjugate vaccine. Nanomedicine.

[51] Niikura K., Matsunaga T., Suzuki T., Kobayashi S., Yamaguchi H., Orba Y., Kawaguchi A., Hasegawa H., Kajino K., Ninomiya T. (2013). Gold Nanoparticles as a Vaccine Platform: Influence of Size and Shape on Immunological Responses in Vitro and in Vivo. ACS Nano.

[52] Webster D.M., Sundaram P., Byrne M.E. (2013). Injectable nanomaterials for drug delivery: Carriers, targeting moieties, and therapeutics. Eur. J. Pharm. Biopharm..

[53] Lin A.Y., Lunsford J., Bear A.S., Young J.K., Eckels P., Luo L., Foster A.E., Drezek R.A. (2013). High-density sub-100-nm peptide-gold nanoparticle complexes improve vaccine presentation by dendritic cells in vitro. Nanoscale Res. Lett..

[54] Cao-Milan R., Liz-Marzan L.M. (2014). Gold nanoparticle conjugates: Recent advances toward clinical applications. Expert Opin. Drug Deliv..

[55] Bolhassani A., Javanzad S., Saleh T., Hashemi M., Aghasadeghi M.R., Sadat S.M. (2014). Polymeric nanoparticles Potent vectors for vaccine delivery targeting cancer and infectious diseases. Hum. Vaccines Immunotherap..

[56] Cruz L.J., Tacken P.J., Zeelenberg I.S., Srinivas M., Bonetto F., Weigelin B., Eich C., de Vries I.J., Figdor C.G. (2014). Tracking Targeted Bimodal Nanovaccines: Immune Responses and Routing in Cells, Tissue, and Whole Organism. Mol. Pharm..

[57] Torres A.G., Gregory A.E., Hatcher C.L., Vinet-Oliphant H., Morici L.A., Titball R.W., Roy C.J. (2015). Protection of non-human primates against glanders with a gold nanoparticle glycoconjugate vaccine. Vaccine.

[58] Tavernaro I., Hartmann S., Sommer L., Hausmann H., Rohner C., Ruehl M., Hoffmann-Roeder A., Schlecht S. (2015). Synthesis of tumor-associated MUC1-glycopeptides and their multivalent presentation by functionalized gold colloids. Org. Biomol. Chem..

[59] Popescu R.C., Grumezescu A.M. (2015). Metal Based Frameworks for Drug Delivery Systems. Curr. Top. Med. Chem..

[60] Zhang X.Y. (2015). Gold Nanoparticles: Recent Advances in the Biomedical Applications. Cell Biochem. Biophys..

[61] Pelliccia M., Andreozzi P., Paulose J., D'Alicarnasso M., Cagno V., Donalisio M., Civra A., Broeckel R.M., Haese N., Silva P.J. (2016). Additives for vaccine storage to improve thermal stability of adenoviruses from hours to months. Nat. Commun..

[62] Chen Y.-S., Hung Y.-C., Lin W.-H., Huang G.S. (2010). Assessment of gold nanoparticles as a size-dependent vaccine carrier for enhancing the antibody response against synthetic foot-and-mouth disease virus peptide. Nanotechnology.

[63] Barhate G.A., Gaikwad S.M., Jadhav S.S., Pokharkar V.B. (2014). Structure function attributes of gold nanoparticle vaccine association: Effect of particle size and association temperature. Int. J.Pharm..

[64] Fernandez T.D., Pearson J.R., Leal M.P., Torres M.J., Blanca M., Mayorga C., Le Guevel X. (2015). Intracellular accumulation and immunological properties of fluorescent gold nanoclusters in human dendritic cells. Biomaterials.

[65] Zhou Q.Q., Zhang Y.L., Du J., Li Y., Zhou Y., Fu Q.X., Zhang J.G., Wang X.H., Zhan L.S. (2016). Different-Sized Gold Nanoparticle Activator/Antigen Increases Dendritic Cells Accumulation in Liver-Draining Lymph Nodes and CD8+T Cell Responses. ACS Nano.

[66] Freivalds J., Kotelovica S., Voronkova T., Ose V., Tars K., Kazaks A. (2014). Yeast-Expressed Bacteriophage-Like Particles for the Packaging of Nanomaterials. Mol. Biotechnol..

[67] Zhang P.P., Chiu Y.C., Tostanoski L.H., Jewell C.M. (2015). Polyelectrolyte Multilayers Assembled Entirely from Immune Signals on Gold Nanoparticle Templates Promote Antigen-Specific T Cell Response. ACS Nano.

[68] Zhang P.P., Andorko J.I., Jewell C.M. (2017). Impact of dose, route, and composition on the immunogenicity of immune polyelectrolyte multilayers delivered on gold templates. Biotechnol. Bioeng..

[69] Wang Y.R., Wang Y., Kang N., Liu Y.L., Shan W.J., Bi S.L., Ren L., Zhuang G.H. (2016). Construction and Immunological Evaluation of CpG-Au@HBc Virus-Like Nanoparticles as a Potential Vaccine. Nanoscale Res. Lett..

[70] Cao Y.H., Ma Y.F., Zhang M.X., Wang H.M., Tu X.L., Shen H., Dai J.W., Guo H.C., Zhang Z.J. (2014). Ultrasmall Graphene Oxide Supported Gold Nanoparticles as Adjuvants Improve Humoral and Cellular Immunity in Mice. Adv. Funct. Mater..

[71] Tao Y., Ju E.G., Li Z.H., Ren J.S., Qu X.G. (2014). Engineered CpG-Antigen Conjugates Protected Gold Nanoclusters as Smart Self- Vaccines for Enhanced Immune Response and Cell Imaging. Adv. Funct. Mater..

[72] Luo Y.H., Wu Z.W., Tsai H.T., Lin S.Y., Lin P.P. (2015). Endotoxin Nanovesicles: Hydrophilic Gold Nanodots Control Supramolecular Lipopolysaccharide Assembly for Modulating Immunological Responses. Nano Lett..

[73] Chen D.X., Weis K.F., Chu Q.L., Erickson C., Endres R., Lively C.R., Osorio J., Payne L.G. (2001). Epidermal powder immunization induces both cytotoxic T-lymphocyte and antibody responses to protein antigens of influenza and hepatitis B viruses. J. Virol..

[74] Dean H.J., Fuller D., Osorio J.E. (2003). Powder and particle-mediated approaches for delivery of DNA and protein vaccines into the epidermis. Comp. Immunol. Microbiol. Infect. Dis..

[75] Madalinski K. (2008). Recent advances in Hepatitis B vaccination. Hepat. B Annu..

[76] Kaurav M., Minz S., Sahu K., Kumar M., Madan J., Pandey R.S. (2016). Nanoparticulate mediated transcutaneous immunization: Myth or reality. Nanomed. Nanotechnol. Biol. Med..

[77] Pissuwan D., Nose K., Kurihara R., Kaneko K., Tahara Y., Kamiya N., Goto M., Katayama Y., Niidome T. (2011). A Solid-in-Oil Dispersion of Gold Nanorods Can Enhance Transdermal Protein Delivery and Skin Vaccination. Small.

[78] Pirmoradi F.N., Pattekar A.V., Linn F., Recht M.I., Volkel A.R., Wang Q., Anderson G.B., Veiseh M., Kjono S., Peeters E. (2015). A microarray MEMS device for biolistic delivery of vaccine and drug powders. Hum. Vaccines Immunotherap..

[79] Crosta P. Cancer: Facts, Causes, Symptoms and Research. http://www.medicalnewstoday.com/info/cancer-oncology.

[80] Asadi N., Davaran S., Panahi Y., Hasanzadeh A., Malakootikhah J., Moafi H.F., Akbarzadeh A. (2017). Application of nanostructured drug delivery systems in immunotherapy of cancer: A review. Artif. Cells Nanomed. Biotechnol..

[81] Coley W.B. (1928). End results in Hodgkin‘s disease and lymphosarcoma treated by the mixed toxins of erysipelas and bacillus prodigiosus, alone or combined with radiation. Ann. Surg..

[82] Mocan T., Matea C., Tabaran F., Iancu C., Orasan R., Mocan L. (2015). In Vitro Administration of Gold Nanoparticles Functionalized with MUC-1 Protein Fragment Generates Anticancer Vaccine Response via Macrophage Activation and Polarization Mechanism. J. Cancer.

[83] Parry A.L., Spain S.G., Ellis J., Davis B.D., Cameron N.R. (2009). Glycopolymer-functionalized gold nanoparticles: A new strategy toward synthetic anticancer vaccines. Abstr. Pap. Am. Chem. Soc..

[84] Brinas R.P., Sundgren A., Maetani M., Abbudayyeh O., Young H.A., Sanford M., Barchi J.J. (2010). Development of a novel cancer vaccine based on multivalent presentation of tumor-associated carbohydrate antigens on gold nanoparticle scaffolds. Abstr. Pap. Am. Chem. Soc..

[85] Brinas R.P., Sundgren A., Sahoo P., Morey S., Rittenhouse-Olson K., Wilding G.E., Deng W., Barchi J.J. (2012). Design and Synthesis of Multifunctional Gold Nanoparticles Bearing Tumor-Associated Glycopeptide Antigens as Potential Cancer Vaccines. Bioconj. Chem..

[86] Lee I.-H., Kwon H.-K., An S., Kim D., Kim S., Yu M.K., Lee J.-H., Lee T.-S., Im S.-H., Jon S. (2012). Imageable antigen-presenting gold nanoparticle vaccines for effective cancer immunotherapy in vivo. Angew. Chem. Int. Ed..

[87] Almeida J.P.M., Figueroa E.R., Drezek R.A. (2014). Gold nanoparticle mediated cancer immunotherapy. Nanomed. Nanotechnol. Biol. Med..

[88] Ahn S., Lee I.H., Kang S., Kim D., Choi M., Saw P.E., Shin E.C., Jon S. (2014). Gold Nanoparticles Displaying Tumor-Associated Self-Antigens as a Potential Vaccine for Cancer Immunotherapy. Adv. Healthc. Mater..

[89] Zhou X., Liu R.H., Qin S., Yu R.L., Fu Y. (2016). Current Status and Future Directions of Nanoparticulate Strategy for Cancer Immunotherapy. Curr. Drug Metab..

[90] Dykman L.A., Staroverov S.A., Bogatyrev V.A., Shchyogolev S.Y. (2010). Gold Nanoparticles as An antigen and as an Adjuvant.

[91] Parry A.L., Clemson N.A., Ellis J., Bernhard S.S.R., Davis B.G., Cameron N.R. (2013). ‘Multicopy Multivalent‘ Glycopolymer-Stabilized Gold Nanoparticles as Potential Synthetic Cancer Vaccines. J. Am. Chem. Soc..

[92] Cai H., Degliangeli F., Palitzsch B., Gerlitzki B., Kunz H., Schmitt E., Fiammengo R., Westerlind U. (2016). Glycopeptide-functionalized gold nanoparticles for antibody induction against the tumor associated mucin-1 glycoprotein. Bioorg. Med. Chem..

[93] Almeida J.P.M., Lin A.Y., Figueroa E.R., Foster A.E., Drezek R.A. (2015). In vivo Gold Nanoparticle Delivery of Peptide Vaccine Induces Anti-Tumor Immune Response in Prophylactic and Therapeutic Tumor Models. Small.

[94] Liu Y., Chen C.Y. (2016). Role of nanotechnology in HIV/AIDS vaccine development. Adv. Drug Deliv. Rev..

[95] Marradi M., Di Gianvincenzo P., Enriquez-Navas P.M., Martinez-Avila O.M., Chiodo F., Yuste E., Angulo J., Penades S. (2011). Gold Nanoparticles Coated with Oligomannosides of HIV-1 Glycoprotein gp120 Mimic the Carbohydrate Epitope of Antibody 2G12. J. Mol. Biol..

[96] Di Gianvincenzo P., Chiodo F., Marradi M., Penades S. (2012). Gold manno-glyconanoparticles for intervening in HIV gp120 carbohydrate-mediated processes. Methods Enzymol..

[97] Glass J.J., Kent S.J., De Rose R. (2016). Enhancing dendritic cell activation and HIV vaccine effectiveness through nanoparticle vaccination. Expert Rev. Vaccines.

[98] Morner A., Jansson M., Bunnik E.M., Scholler J., Vaughan R., Wang Y., Montefiori D.C., Otting N., Bontrop R., Bergmeier L.A. (2011). Immunization with Recombinant HLA Classes I and II, HIV-1 gp140, and SIV p27 Elicits Protection against Heterologous SHIV Infection in Rhesus Macaques. J. Virol..

[99] Abia I., Peng T.Y., Mains S., Pohl N. (2013). Design and synthesis of thiol-terminated oligosaccharides for attachment on gold nanoparticles: Toward the development of an HIV vaccine. Abstr. Pap. Am. Chem. Soc..

[100] Chiodo F., Enriquez-Navas P.M., Angulo J., Marradi M., Penades S. (2015). Assembling different antennas of the gp120 high mannose-type glycans on gold nanoparticles provides superior binding to the anti-HIV antibody 2G12 than the individual antennas. Carbohydr. Res..

[101] Di Gianvincenzo P., Calvo J., Perez S., Alvarez A., Bedoya L.M., Alcami J., Penades S. (2015). Negatively Charged Glyconanoparticles Modulate and Stabilize the Secondary Structures of a gp120 V3 Loop Peptide: Toward Fully Synthetic HIV Vaccine Candidates. Bioconj. Chem..

[102] Xu L., Liu Y., Chen Z., Li W., Liu Y., Wang L., Liu Y., Wu X., Ji Y., Zhao Y. (2012). Surface-Engineered Gold Nanorods: Promising DNA Vaccine Adjuvant for HIV-1 Treatment. Nano Lett..

[103] Demenev V.A., Shchinova M.A., Ivanov L.I., Vorobeva R.N., Zdanovskaia N.I., Nebaikina N.V. (1996). Perfection of methodical approaches to designing vaccines against tick-borne encephalitis. Vopr. Virusol..

[104] Zhao Z.J., Wakita T., Yasui K. (2003). Inoculation of plasmids encoding Japanese encephalitis virus PrM-E proteins with colloidal gold elicits a protective immune response in BALB/c mice. J. Virol..

[105] Yuan R., Zhang L.Y., Li Q.F., Chai Y.Q., Cao S.R. (2005). A label-free amperometric immunosenor based on multi-layer assembly of polymerized o-phenylenediamine and gold nanoparticles for determination of Japanese B encephalitis vaccine. Anal. Chim. Acta.

[106] Zhang L.Y., Yuan R., Chai Y.Q., Chen S.H., Wang N., Zhu Q. (2006). Layer-by-layer self-assembly of films of nano-Au and Co(bpy)(3)(3+) for the determination of Japanese B encephalitis vaccine. Biochem. Eng. J..

[107] Zhang L., Widera G., Bleecher S., Zaharoff D.A., Mossop B., Rabussay D. (2003). Accelerated immune response to DNA vaccines. DNA Cell Biol..

[108] Zhang L., Widera G., Rabussay D. (2004). Enhancement of the effectiveness of electroporation-augmented cutaneous DNA vaccination by a particulate adjuvant. Bioelectrochemistry.

[109] Pilling A.M., Harman R.M., Jones S.A., McCormack N.A.M., Lavender D., Haworth R. (2002). The assessment of local tolerance, acute toxicity, and DNA biodistribution following particle-mediated delivery of a DNA vaccine to minipigs. Toxicol. Pathol..

[110] Draz M.S., Wang Y.J., Chen F.F., Xu Y.H., Shafiee H. (2017). Electrically Oscillating Plasmonic Nanoparticles for Enhanced DNA Vaccination against Hepatitis C Virus. Adv. Funct. Mater..

[111] Wang H., Ding Y.P., Su S.S., Meng D.J., Mujeeb A., Wu Y., Nie G.J. (2016). Assembly of hepatitis E vaccine by ‘in situ‘ growth of gold clusters as nano-adjuvants: An efficient way to enhance the immune responses of vaccination. Nanoscale Horiz..

[112] Dykman L.A., Staroverov S.A., Mezhenny P.V., Fomin A.S., Kozlov S.V., Volkov A.A., Laskavy V.N., Shchyogolev S.Y. (2015). Use of a synthetic foot-and-mouth disease virus peptide conjugated to gold nanoparticles for enhancing immunological response. Gold Bull..

[113] Pokharkar V., Bhumkar D., Suresh K., Shinde Y., Gairola S., Jadhav S.S. (2011). Gold Nanoparticles as a Potential Carrier for Transmucosal Vaccine Delivery. J. Biomed. Nanotechnol..

[114] Barhate G., Gautam M., Gairola S., Jadhav S., Pokharkar V. (2014). Enhanced Mucosal Immune Responses Against Tetanus Toxoid Using Novel Delivery System Comprised of Chitosan-Functionalized Gold Nanoparticles and Botanical Adjuvant: Characterization, Immunogenicity, and Stability Assessment. J. Pharm. Sci..

[115] Barhate G., Gautam M., Gairola S., Jadhav S., Pokharkar V. (2013). Quillaja saponaria extract as mucosal adjuvant with chitosan functionalized gold nanoparticles for mucosal vaccine delivery: Stability and immunoefficiency studies. Int. J. Pharm..

[116] Stone J., Thornburg N.J., Blum D.L., Kuhn S.J., Wright D.W., Crowe J.E. (2013). Gold nanorod vaccine for respiratory syncytial virus. Nanotechnology.

[117] Tao W.Q., Ziemer K.S., Gill H.S. (2014). Gold nanoparticle-M2e conjugate coformulated with CpG induces protective immunity against influenza A virus. Nanomedicine.

[118] Tao W.Q., Gill H.S. (2015). M2e-immobilized gold nanoparticles as influenza A vaccine: Role of soluble M2e and longevity of protection. Vaccine.

[119] Staroverov S.A., Vidyasheva I.V., Gabalov K.P., Vasilenko O.A., Laskavyi V.N., Dykman L.A. (2011). Immunostimulatory Effect of Gold Nanoparticles Conjugated with Transmissible Gastroenteritis Virus. Bull. Exp. Biol. Med..

[120] Calderon-Gonzalez R., Marradi M., Garcia I., Petrovsky N., Alvarez-Dominguez C. (2015). Novel nanoparticle vaccines for Listeriosis. Hum. Vaccines Immunotherapeut..

[121] Rodriguez-Del Rio E., Marradi M., Calderon-Gonzalez R., Frande-Cabanes E., Penadés S., Petrovsky N., Alvarez-Dominguez C. (2015). A gold glyco-nanoparticle carrying a listeriolysin O peptide and formulated with Advax™ delta inulin adjuvant induces robust T-cell protection against listeria infection. Vaccine.

[122] Calderon-Gonzalez R., Teran-Navarro H., Frande-Cabanes E., Ferrandez-Fernandez E., Freire J., Penades S., Marradi M., Garcia I., Gomez-Roman J., Yanez-Diaz S. (2016). Pregnancy Vaccination with Gold Glyco-Nanoparticles Carrying Listeria monocytogenes Peptides Protects against Listeriosis and Brain- and Cutaneous-Associated Morbidities. Nanomaterials.

[123] Yang Y.S.S., Atukorale P.U., Moynihan K.D., Bekdemir A., Rakhra K., Tang L., Stellacci F., Irvine D.J. (2017). High-throughput quantitation of inorganic nanoparticle biodistribution at the single-cell level using mass cytometry. Nat. Commun..

[124] Gregory A.E., Williamson E.D., Prior J.L., Butcher W.A., Thompson I.J., Shaw A.M., Titball R.W. (2012). Conjugation of Y. pestis F1-antigen to gold nanoparticles improves immunogenicity. Vaccine.

[125] Kumar R., Ray P.C., Datta D., Bansal G.P., Angov E., Kumar N. (2015). Nanovaccines for malaria using Plasmodium falciparum antigen Pfs25 attached gold nanoparticles. Vaccine.

[126] Dakterzada F., Mobarez A.M., Roudkenar M.H., Mohsenifar A. (2016). Induction of humoral immune response against Pseudomonas aeruginosa flagellin(1-161) using gold nanoparticles as an adjuvant. Vaccine.

[127] Chen H.W., Huang C.Y., Lin S.Y., Fang Z.S., Hsu C.H., Lin J.C., Chen Y.I., Yao B.Y., Hu C.M.J. (2016). Synthetic virus-like particles prepared via protein corona formation enable effective vaccination in an avian model of coronavirus infection. Biomaterials.

[128] Gregory A.E., Judy B.M., Qazi O., Blumentritt C.A., Brown K.A., Shaw A.M., Torres A.G., Titball R.W. (2015). A gold nanoparticle-linked glycoconjugate vaccine against Burkholderia mallei. Nanomed. Nanotechnol. Biol. Med..

[129] Zhou Y., Jiang N., Ma J., Fan Y.D., Zhang L.L., Xu J., Zeng L.B. (2015). Protective immunity in gibel carp, Carassius gibelio of the truncated proteins of cyprinid herpesvirus 2 expressed in Pichia pastoris. Fish Shellfish Immunol..

[130] Ocampo M., Rodriguez D.C., Rodriguez J., Bermudez M., Munoz C.M., Patarroyo M.A., Patarroyo M.E. (2013). Rv1268c protein peptide inhibiting Mycobacterium tuberculosis H37Rv entry to target cells. Bioorg. Med. Chem..

[131] Paul A.M., Shi Y.L., Acharya D., Douglas J.R., Cooley A., Anderson J.F., Huang F.Q., Bai F.W. (2014). Delivery of antiviral small interfering RNA with gold nanoparticles inhibits dengue virus infection in vitro. J. Gen. Virol..

[132] Gao W.W., Fang R.H., Thamphiwatana S., Luk B.T., Li J.M., Angsantikul P., Zhang Q.Z., Hu C.M.J., Zhang L.F. (2015). Modulating Antibacterial Immunity via Bacterial Membrane-Coated Nanoparticles. Nano Lett..

[133] DeRussy B.M., Aylward M.A., Fan Z., Ray P.C., Tandon R. (2014). Inhibition of cytomegalovirus infection and photothermolysis of infected cells using bioconjugated gold nanoparticles. Sci. Rep..

[134] Zaffran M., Vandelaer J., Kristensen D., Melgaard B., Yadav P., Antwi-Agyei K.O., Lasher H. (2013). The imperative for stronger vaccine supply and logistics systems. Vaccine.

